# Behavior, Intake, Digestion and Milk Yield of Early Lactation Holstein Dairy Cows with Two Levels of Environmental Exposure and Feeding Strategy

**DOI:** 10.3390/ani14131905

**Published:** 2024-06-27

**Authors:** Maria Noel Méndez, Nadia Swanepoel, Peter H. Robinson, Victoria Pons, Alejandra Jasinsky, Maria de Lourdes Adrien, Pablo Chilibroste

**Affiliations:** 1 Departamento de Ciencias Veterinarias y Agrarias, Facultad de Veterinaria, Universidad de la República, Paysandú 60000, Uruguay; 2 Department of Animal Science, University of California, Davis, CA 95616, USA; 3 Departamento de Producción Animal y Pasturas, Facultad de Agronomía, Universidad de la República, Paysandú 60000, Uruguay; pchili@fagro.edu.uy

**Keywords:** grazing, mixed ration, confinement, behavior, microbial crude protein, milk yield

## Abstract

**Simple Summary:**

Higher stocking rates and supplemental feed intake in intensified pasture-based dairy systems (mixed systems) result in more confinement time. Thus, housing design is more important in minimizing environmental effects on animal performance. In addition, some Uruguayan dairy farmers are replacing low-input, but complex, mixed systems with total confinement (i.e., total mixed-ration systems) to better balance animal energy demand and exert more control over environmental exposure in order to optimize milk production. This study aims to determine the magnitude of the effects of implementing a partially confined grazing system with access to outdoor soil-bedded pens or a compost-bedded pack barn and a fully confined compost-bedded pack barn system (both of the latter with cooling capacity) on behavior, intake, nutrient utilization and microbial outflow, as well as milk production and composition, during early lactation in two calving seasons. The results demonstrate that under our relatively benign weather conditions, the cows in the outdoor soil-bedded system adapted some behaviors to compensate for their poorer living conditions but had a similar nutrient intake, utilization and microbial crude protein synthesis than the cows in the enhanced confinement systems. The fully confined cows greatly outperformed those in the other systems, enhancing milk production by 20–35% and feed efficiency by 8–18%, with no impact on milk component levels.

**Abstract:**

Eighty-four autumn (ACS, *n* = 45)- and spring (SCS, *n* = 39)-calved multiparous early lactation Holstein cows were assigned to groups of either: (a) grazing + mixed ration (MR) during partial confinement in outdoor soil-bedded pens with shade (OD-GRZ); (b) grazing + MR during partial confinement in a compost-bedded pack barn with cooling (CB-GRZ); or (c) total confinement fed a totally mixed ration (CB-TMR) in a compost-bedded pack barn. Data were analyzed using the SAS MIXED procedure with significance at *p* ≤ 0.05. In both seasons, despite behavioral differences (*p* < 0.05) between the OD-GRZ and CB-GRZ groups (i.e., standing, first grazing meal length, bite rate), the milk and component yields, DM intake, microbial CP output (MCP) and NE efficiency were unaffected by the housing conditions, possibly due to mild weather conditions. The milk yield was substantially higher in the CB-TMR group versus the OD-TMR and CB-TMR groups (*p* < 0.01) in both ACS (~35%) and SCS (~20%) despite there being no intake differences, without any impact on milk component levels. In ACS, this was associated with a higher MCP, likely due to the higher nutritional value of TMR compared to pasture, which was not the case in SCS. In conclusion, the OD-GRZ group achieved the same milk production as the CB-GRZ group through behavior adaptation, under mild weather conditions, in both calving seasons. The CB-TMR group outperformed the grazing systems in both calving seasons, regardless of the MCP.

## 1. Introduction

The sustainable intensification of pasture-based dairy farms implies a balanced increase in individual cow milk production and stocking rates in order to increase non-pasture feed intake (supplement, i.e., mixed ration; MR) without losing the potential to harvest home-grown forages by cows, thus allowing for low production costs and facilitating international competitiveness [1]. In this sense, Ortega et al. [2] reported that farmlets with a stocking rate of 1.5 cows per hectare had access to two grazing turns per day for 56% of the lactation days and one grazing turn per day for 40% of the lactation days. In contrast, farmlets with a stocking rate of 2.0 cows per hectare had access to two grazing turns per day for 42% of the lactation days and one grazing turn per day for 28% of the lactation days. The higher stocking rates of mixed systems (i.e., pasture + supplement; **MS**) result in more confinement time at feeders or in resting areas when the pasture is inaccessible (i.e., during heavy rains or when there is a low herbage mass) [2]. Hence, the design of housing facilities is important in intensified MS dairy farms. 

Although higher levels of directly harvested forage dry matter intake (**DMI**) make systems more competitive, Uruguayan systems exploring the borders of stocking rates (mostly open-door, dry-lot) face the problem of not being prepared, largely due to poor infrastructure and management, to withstand long and/or repeated confinement periods, thereby exposing cows to extreme weather conditions such as high temperature, humidity and solar radiation or mud after heavy rainfall [3]. In this sense, the efficiency of nutrient use for milk production depends not only on the composition of the diet, cow genotype and physiological state but also on the weather and its influence on cow behaviors [4]. Housing conditions determine cow comfort as well as their quantity and quality of rest, which in turn define cow behaviors and activities [5,6], especially when they have to graze, as is the case in MS. Unmet needs for rest, lying and rumination affect cows’ motivation to search for grazing and harvest [7,8], altering their ingestive patterns, ruminal conditions and digestive processes [9]. These situations can occur when cows are exposed to extreme weather, as the facilities provided may not mitigate the environmental effects, with cows having to regulate their body temperature through behavior and physiological changes [10,11], or when conditions are too wet to stimulate cows to lie down, such as when there is mud in resting areas [8,12], as can be the case in the intensive Uruguayan pastoral systems. Thus, it becomes imperative to determine the extent to which these conditions limit cow performance. The first approach of our group to addressing this problem revealed that enhanced housing facilities allowed for better performance during specific periods of environmental stress or even during the whole lactation period, dependent on the lactation period when the cows faced climatic challenges, which is linked to the calving season [13]. As a second step, it was necessary to determine which mechanisms cows use to compensate for impaired environmental conditions and/or how inefficiencies occur when they fail to overcome them. 

In Uruguay, 46% of annual calvings are concentrated in the autumn and 24% in the spring [14]. The distribution of calvings has a direct effect on the instantaneous stocking rate of the system and the total feed demand in relation to supply, which determines the need for supplementation to compensate for insufficient pasture intake. In this sense, rumen utilization and productive performance depend on the level of inclusion and quality of each component of the diet [15]. On the other hand, feeding management in the first weeks after parturition is crucial in establishing the milk production potential, metabolic health and overall productivity of the cow throughout its lactation cycle [16,17,18], so nutritional and herd management in early lactation is a key point. During the fall–winter period, the pasture growth rate and available pasture in the rotation are minimal, so supplementation is maximized. At the same time, excessive rainfall, either in quantity or frequency, can impede pasture access and compromise animal welfare. In the spring, pasture use is maximized while environmental factors such as heat stress are present, which could affect productive performance during the most demanding period of the productive cycle [19,20].

In addition, some Uruguayan dairy farmers are replacing low-input, but managerially complex, MS with total confinement (i.e., total mixed-ration—TMR—systems) to overcome the gap between feed demand and supply and to exert more control over environmental exposure in order to optimize milk production [15,21,22]. Although this issue has been previously studied [23,24,25], it is necessary to quantify the productivity gap between the two feeding systems (MS vs. TMR) but with different levels of environmental exposure under local environmental conditions and with available feed. 

Our goal was to study ingestive behavior, DM intake and ruminal microbial CP synthesis as factors associated with feed efficiency and milk yield in two mixed-management systems (i.e., cows grazed in pasture and those fed a mixed ration) with high or low environmental exposure compared to a reference TMR system in autumn- (ACS) and spring-calved (SCS) early lactation cows. 

## 2. Materials and Methods

### 2.1. Cows and Experimental Design

Two experiments with autumn- and spring-calved Holstein dairy cows were conducted at the Estación Experimental Dr. M. A. Cassinoni (EEMAC) of the Facultad de Agronomía (Paysandú, Uruguay) of Universidad de la República (UdelaR). The autumn-calved cows were as follows: Experiment 1: *n* = 45, 2.9 ± 1.4 lactations, 654 ± 99 kg body weight (BW), calving dates of 16 March 2019 ± 10 days at 3.0 ± 0.27 points of BCS. The spring-calved cows were as follows: Experiment 2, *n* = 39, 2.7 ± 0.9 lactations, 624 ± 61 kg BW, calving dates of 9 August 2019 ± 13 days at 2.8 ± 0.22 points of BCS. All cows were managed similarly during their dry and prepartum periods when they were confined and fed a prepartum TMR for 3 weeks before their expected calving date. The cows were blocked by BW, expected calving date, lactation number and pre-calving and body condition score (BCS) according to [26] before random assignment to treatments and grouping into pens of 4 cows each (i.e., 16 cows/treatment). The cows were assigned to their corresponding treatments immediately after calving. The sampling periods consisted of 5 days of measurements and samplings during week 6 of the study for ACS (44 ± 10 days in milk) and week 8 for SCS (50 ± 13 days in milk), which corresponded to late April and late September, respectively. Some cows were removed from the experiment due to calving or postpartum illnesses and were replaced with non-study cows in order to maintain equal conditions in all pens. The experimental protocol was evaluated and approved by the Comisión de Ética en el Uso de Animales de la Facultad de Agronomía (ID 682- Exp020300-000602-18), UdelaR (Montevideo, Uruguay).

In both experiments, the cows were assigned to one of three treatments, which consisted of: (1) high environmental exposure in a mixed-management system (MS: i.e., outdoor soil-bedded pens with shade structures) when not out to pasture (OD-GRZ); (2) low environmental exposure in an MS (i.e., compost-bedded pack barn with cooling capacity) when not out to pasture (CB-GRZ); or (3) a totally confined system with cows in the same facilities as the CB-GRZ group but fed a TMR twice daily ad libitum (CB-TMR, similar chemical composition to MS). 

### 2.2. Management and Feeding

This study was part of a larger experiment [13] in which cow performance (i.e., DM intake, milk production and composition, BW, BCS, energy metabolism) were measured over a full lactation to evaluate cows’ treatment responses (from 0 to 290 days in milk, which corresponded to from March 2019 to January 2020 for ACS cows and from August 2019 to May 2020 for SCS cows).

The OD-GRZ cows were confined in outdoor soil-bedded pens (48 m^2^ per cow) with shade structures (nylon roof at 4.5 m height with a slope of 15%, 4.8 m^2^ per cow). The pens had a slight slope for water and manure runoff and were divided in half, and they were alternately occupied according to soil moisture and surface deterioration. Feeders were located at an end of the paddocks at a feeding area of 10 m^2^/cow, and they had a length of 1.10 m/cow (for a detailed description of these facilities, see [27]).

The CB-TMR and CB-GRZ cows were confined in a fully roofed compost-bedded pack barn (13.5 m^2^/cow) with a concrete floor (6.7 m^2^/cow) with cooling capacity (i.e., fans and with sprinklers fitted with an automatic operation, over 25 °C, of 3 min on and 10 min off. Surface compost was removed twice daily with a chisel plough to remove water vapor, limit oxygen entry and maintain small homogeneous particles. The temperature and humidity of the compost bed were assessed weekly, with new material (i.e., wood chips, rice husks) added every 20 days. The concrete feeding area was cleaned thrice weekly by a tractor-mounted rubber scraper. The feeders were inside the barn but separated from the composted area by a concrete cow standing area (area: 6.7 m^2^/cow; length; 0.75 m/cow). 

The cows were confined in groups of 4 in separate pens when in pens, whereas when outside, they were co-mingled. Water was available ad libitum to all cows by automatic waterers. The cows were milked twice daily at 3:00 and 16:00 h in a milk parlor 100 m from the pens. The TMR fed to the CB-TMR cows was also the mixed ration (MR) fed to the grazing cows but at lower levels, depending on the available pasture. Due to changes in the availability of the stored feed, the ACS and SCS MRs differed in the available conserved forage, which consisted of corn and ryegrass silage for the MR in the ACS experiment and sorghum silage plus fescue hay in the SCS experiment. The diets were formulated based on the guidelines in [28] for 620 kg cows producing 45 L/d of 4% fat-corrected milk. In the MS group, the MR was a pasture complement to achieve the desired DM intake. The ingredients, chemical composition and calculated nutritional value of the TMR/MR are shown in Table 1.

The OD-GRZ and CB-GRZ groups were high-stocking-rate systems (i.e., 2.5 lactating cows and/or 1550 kg BW/ha of grazing platform). The cows grazed between 7:00 and 14:00 h in grazing plots, rotated weekly. The cows with grazing in their treatment accessed various grazing plots with similar herbage allowance (HA). The cows in the MS in ACS grazed on a 2^nd^-year permanent sward composed of Tall Fescue (*Lolium arundinaceum* (Schreb.) Darbysh.)) + Birdsfoot Trefoil (*Lotus corniculatus* L.), and the cows in the SCS MS grazed on a 1st-year permanent sward composed of Lucerne (*Medicago sativa* L.) + Orchard Grass (*Dactylis glomerata* L.). Herbage mass (HM) was determined using a double sampling technique [29], where 5 scale points of biomass availability representative of the field were selected and three replicates of each were cut in the field. The forage management and its nutritional value during the sample periods are shown in Table 2.

### 2.3. Data collection, Measurements and Estimates

Climatic conditions (i.e., ambient temperature, relative humidity, wind speed, rain) during the sampling periods were obtained from the meteorological agency of the experimental station. Heat stress was determined by a temperature humidity index (**THI**) as follows:

(1.8 × ET + 32) − (0.55 × RH/100) × (1.8 × ET − 26)

where ET is the environmental temperature and RH is the relative humidity [30]. 

Individual cow milk production was recorded at each milking during fecal sampling days. Milk samples were collected to determine the fat, crude protein (**CP**) and lactose contents (MilkoScan FossElectric FT2^®^). Milk (L) was converted to a standard of 40 g/L fat and 33 g/L protein (kg, fat-/protein-corrected milk, **FPCM**) using the following formula:

(0.337 × milk production, kg) + (1.16 × fat in g/L + (0.60 × protein in g/L)

according to [31] in order to calculate the feed efficiency (kg FPCM/kg DMI). 

The energy retained in the milk (Mcal NE/cow/day, ERM) was calculated as follows:

(0.929 × fat in g/L + 0.547 × protein in g/L + 0.395 × lactose in g/L) × milk yield, L/d

The amounts of MR offered and refused per pen were recorded daily during the sampling periods in order to determine the DMI. Refused feed was removed and weighed prior to the morning feeding during the sampling periods. Pasture samples were collected every hour by hand clipping, according to [32], using two cows per treatment during scan sampling, which occurred during the grazing sessions on days 1, 3 and 5 of each feces collection period. Each pasture sample was composed of 42 subsamples. The samples were collected and stored at −20 °C. The pasture and TMR/MR samples were weighed and dried in a forced air oven at 60 °C for 48 h. The samples were reweighed after drying to determine the DM content and finally ground through a 1 mm sieve for chemical analysis and in vitro gas production. The mixed-ration samples per pen were pooled to create a composite sample for each treatment. Chemical analysis consisted of the DM, ash (to calculate the organic matter content; OM) and CP as well as neutral detergent (aNDFom) and acid detergent fiber (ADFom) according to [33]. The NDF used α-amylase, and both were assayed using an ANKOM200 Fiber Analyzer (ANKOM Tech. Corp., Fairport, NY, USA). Total N for CP calculation was calculated using the Kjeldahl method [34]. 

In vitro gas production was used to determine the feed nutritional value (i.e., OM digestibility, net energy, fermentation kinetics). Approximately 200 mg of DM of each feed composite sample was incubated in duplicate in rumen fluid in glass syringes following the procedures of [35]. Rumen fluid was collected from 2 donor non-lactating dairy cows fed a diet containing 500 g/kg hay and 500 g/kg concentrate twice daily at a level sufficient to meet their estimated maintenance NE requirements. The syringes were pre warmed at 39 °C before injection of 10 mL of rumen fluid and 20 mL of reducing agent/buffer mineral medium mixture into each syringe, followed by incubation in a water bath at 39 °C. Gas production was recorded at 2, 4, 6, 8, 24, 30, 48, 72 and 96 h of incubation. The gas values were corrected for blank incubation in order to correct for gas production from the fermentation of residual feed in the rumen fluid and for the inter-run standard (i.e., an alfalfa hay standard with a known gas production history) in order to standardize the gas readings among runs. Values were converted to mL gas/g OM. 

**Figure 1:**
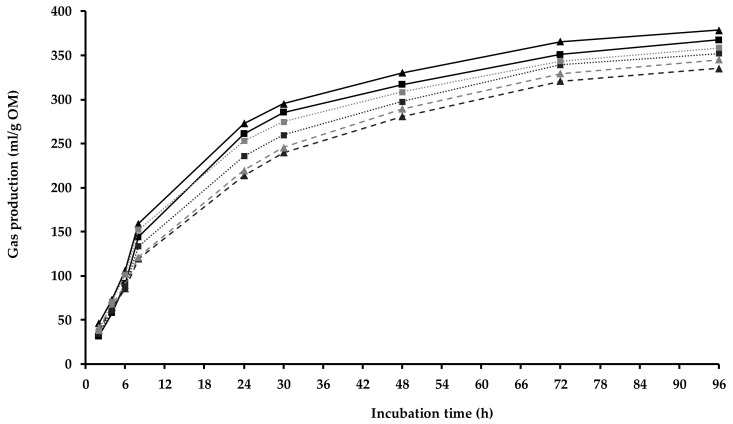
Gas production in mixed diet (continuous line) and pasture (dashed lines) offered to autumn (triangles, ACS)- and spring (squares, SCS)-calving-season cows with low (dark gray, CB-GRZ) or high (light gray, OD-GRZ) environmental exposure.

The cumulative gas production data were fitted to the model of [36] as follows:

y = a + b (1 − *e*^−ct^)

where y is the gas produced at time ‘t’, a is the gas produced (mL) from the immediately soluble fraction, b is the gas producted (mL) from the insoluble fraction (mL), c is the gas production rate constant for the insoluble fraction b, a + b is the potential gas production (mL) and t is the incubation time (h). There was no lag term used in the model, as the gas production was essentially instantaneous (Figure 1), and the ‘a’ values are not reported as they were essentially zero.

The energy value of the TMR/MR and pastures was calculated from the amount of gas produced (GP) at 24 h of incubation and the ether extract (EE) content, according to the following [37]:

ENL (Mcal/kg DM) = 0.689 + 0.0134GP_24_ + 0.0771EE

The total DMI per cow in the mixed systems was estimated with a double marker technique, which consisted of an indigestible external marker (i.e., chromium oxide, Cr_2_O_3_) to estimate the fecal output [38,39] and an internal marker (i.e., acid-insoluble ash) to estimate the DM digestibility [40]. Fecal output was estimated as Cr intake/Cr concentration in the feces. For this purpose, each cow received an oral bolus of 7.5 g of Cr_2_O_3_ at each milking (chromium III oxide, G-105M, 98% purity; Ferro Colombia S.A.S., Colombia) for 12 days, and fecal samples were collected from the rectum for the last 5 days, when the cows returned from AM and PM milking. The cows were observed after dosing to ensure no bolus regurgitation. All the samples were preserved at −20 °C. The samples were dried in a forced air oven at 60 °C to a constant weight and then milled through a 1 mm screen. Subsamples from each cow, day and shift were used to generate a composite sample by cow and shift for analysis. The chromium content was determined according to [41]. The AIA contents of the pasture, TMR/MR and feces were determined according to [42]. The DM digestibility was estimated as follows:

(g AIA/kg DM feces − g AIA/kg MR)/(g AIA/kg DM feces).

Finally, the intake (kg DM/cow/day) was determined as reported by [43] as follows:

fecal output/(1 − diet DM digestibility)

The daily pasture DM intake per cow was estimated as the difference between the total DMI and DMI of the MR. 

Behavioral evaluation was conducted during the fecal sampling periods in both MS (i.e., CB-GRZ and OD-GRZ). The activity pattern of each cow was recorded from direct real-time observation using instantaneous scan sampling on days 2, 4 and 6 of the weekly grazing paddock occupation (day 1, 3 and 5 of the sampling period). For each day, scan recordings were conducted every 10 min throughout grazing (07:00 to 14:00 h) and part of confinement (16:00 to 23:00 h). Access to the offered feed (i.e., pasture and TMR/MR, respectively) determined the start time. In each scan, the cows were as recorded as eating (i.e., grazing or standing with her head in the feeder during confinement), ruminating, lying, standing or other (i.e., drinking, walking, allogrooming). Ruminating vs. eating as well as lying vs. standing were mutually exclusive, but ruminating and lying and ruminating and standing could be simultaneous. During grazing observations, bites/minute were counted, if the cows were eating, in order to determine the bite rate (BR). It was assumed that each eating occurred over the entire 10 min observation in order to determine the first grazing meal length (FGML). Data are presented as the probability of the cows eating, ruminating, lying and standing during the first 90 min, as an expected length for the first active grazing bout [44], and during the total time at grazing or confinement.

The allantoin (AL) content, a derivative of absorbed microbial nucleic acid purines, was determined in urine samples according to Chen and Gomes [45] as an indirect measure to estimate the ruminal microbial CP (MCP) output to the duodenum. Individual urine samples were collected following the fecal sampling days over three consecutive days at six times (i.e., 3:00, 7:00, 11:00, 15:00, 19:00 and 23:00 h), as suggested by [46], in order to obtain a ‘super-sample’ per cow that included the within-day variation. Urine was collected by manual urinary bladder compression by perineal massage into 100 mL flasks with 5 mL of hydrochloric acid (6 N HCl), and the pH was measured prior to freezing at −20 °C to ensure that all values were below 5.0 in order to prevent bacterial destruction of the AL. These frozen samples were later thawed, and aliquots of 7 mL were combined with 1 mL of HCl and diluted with 25 mL of deionized water to a total of 33 mL and re-frozen. Finally, these urine samples were thawed and centrifuged at 1200× *g* for 15 min at 20 to 22 °C and diluted 60 times in order to fit the standard curve. Each sample was analyzed in duplicate. Standards of increasing concentrations (i.e., 20, 40, 60, 80 and100 mg AL/L) were run at the start and end of each run to generate the reference curve. The AL values were corrected for the blank and an inter-run standard in duplicate according to [47,48]. Analytical results were corrected by days of frozen storage (i.e., by microbial destruction during storage) using an equation obtained through analyzing a group of samples repeatedly over the time of storage time as follows:

y = −0.082ln(x) + 0.8954 (r^2^ = 0.9992)

where y is a correction factor depending on x, which is the number of days of pre-dilution. 

In order to estimate the total urinary AL output and ruminal MCP flow to the small intestine, the urine volume was determined by measuring the urine creatinine content, a metabolite of phospho-creatine (energy storage in muscle) that is excreted at a relatively constant rate by the kidneys, according to [46], using a commercial colorimetric assay kit (Item No. 500701, © Cayman Chemical Company, Ann Arbor, MI, USA). Samples were also corrected by blanks, standards and days prior to dilution as follows:

y = −0.0002x^2^ − 0.0246x + 99.999 (r^2^ = 0.9985)

where y is a correction factor depending on x, which is the number of days of pre-dilution. 

### 2.4. Statistical Analysis 

Data were analyzed using the MIXED procedure of SAS (SAS Institute Inc., Cary, NC, USA) using the following model:

Y_ij_ = µ + T_i_ + e_ijk_

where Y_ij_ is the response variable, T_i_ is the treatment and e_ijk_ is the residual error. Cow was accepted as the experimental unit for milk production and composition, behavior and microbial CP yield, with pen as a random effect. For the TMR and pasture DM intake, pen was the experimental unit. The model (co)variance structure was AR (1), selected based on the smallest Bayesian information criterion (BIC) value. For all variables except behavior, normality was assumed and tested. For behavior, a binomial distribution was assumed and tested. The probability of cows grazing, ruminating or being engaged in other activities was calculated using a mixed model that included the fixed effects of the treatments and the residual error. Mean comparisons were performed by Tukey–Kramer’s analysis. Mean differences were considered significant if *p* ≤ 0.05. Results are shown as least square means ± standard error of the mean (SEM). 

## 3. Results

The daily mean temperatures during the sampling weeks were 16.4 and 19.0 °C, with a THI daily mean of 61 (0% of daily time > 72) and 64 (19% of daily time > 72) in ACS and SCS, respectively. The wind speed averaged 3.1 and 4.3 km/h, and the accumulated rain was 12 and 2 mm, respectively, for each calving season, with a total rainfall of 71 and 37 mm in April and September 2019 (ACS and SCS, respectively. Please refer to Supplementary Table S1). 

The ACS MR/TMR included a commercial feed concentrate plus corn and ryegrass silage, while the SCS MR/TMR was the same concentrate plus sorghum silage and fescue hay (Table 1). The MR/TMR diets differed numerically in DM content but had a similar chemical composition, estimated NE concentration, fermentability (i.e., mL gas produced/200 mg DM at 24 h of incubation, Figure 1), mL gas produced/g OM at 30 h of incubation, potentially degraded fraction (i.e., ‘b’) and rate of gas appearance (i.e., ‘c’). The OM digestibility was judged as higher in the ACS vs. SCS MR/TMR.

**Table 1:** Composition and predicted nutritional value of the mixed diets fed to autumn (ACS)- and spring (SCS)-calved cows.

			ACS	SCS
Ingredient (g/kg DM)				
	Forage			
		Corn silage	246	-
		Sorghum silage	-	375
		Ryegrass silage	214	-
		Fescue hay	-	65
	Concentrate mixture ^1^		540	560
Dry matter (DM; g/kg)			434	559
Nutrient (g/kg DM)				
		Crude protein	159	165
		Neutral detergent fiber	331	295
		Acid detergent fiber	165	135
		Ether extract	38	38
		Starch	25	29
NE_L_ (Mcal/kg OM) ^2^			1.66	1.62
Gas production ^3^				
		24 h (mL/200 mg DM)	51	47
		30 h (mL/g OM) ^3^	295	285
Rumen kinetics ^4^				
		b (mg/g OM)	367	360
		c (h^−1^)	0.058	0.053
OM digestibility (g/kg) ^5^			609	582

^1^ Based on ground corn grain, wheat bran, soybean meal, sunflower meal, cottonseed meal, canola meal, rumen inert fat, urea, yeast and minerals. ^2^ Net energy of lactation, determined from accumulated gas production at 24 h (mL/200 mg DM) and ether extract content, as described by [38]. ^3^ In vitro following the Menke and Steingass [36] procedure. ^4^ According to the model of Ørskov and McDonald [37]. ^5^ Apparent, in whole tract, according to Menke and Steingass [36].

Both the ACS MS cow treatments grazed a permanent sward with a similar chemical composition, NE concentration, fermentability and OM digestibility, although the HM was lower in the CB-GRZ group than in the OD-GRZ group (Table 2). For the SCS group, the MS had similar HA and DM contents, though there was an apparent higher OM digestibility in the OD-GRZ pasture.

**Table 2:** Characteristics of pasture grazed by autumn (ACS)- and spring (SCS)-calved cows with low (CB-GRZ) or high (OD-GRZ) environmental exposure.

		ACS		SCS	
		CB-GRZ	OD-GRZ	CB-GRZ	OD-GRZ
Herbage mass (kg DM/ha)		1550	2200	3400	2500
Herbage allowance (kg DM/cow/day)		18	18	23	21
Herbage dry matter (g/kg)		238	227	279	277
Nutrient (g/kg DM)					
	Crude protein	149	147	156	139
	Neutral detergent fiber	517	481	610	548
	Acid detergent fiber	246	241	315	262
	Ether extract	42	40	31	39
	Ash	111	110	108	114
NE_L_ (Mcal/kg OM) ^1^		1.53	1.53	1.49	1.60
Gas production ^2^					
	24 h (mL/200 mg DM)	39	40	42	45
	30 h (mL/g OM)	240	245	260	275
Rumen kinetics ^3^					
	b (mg/g OM)	329	338	346	345
	c (h^−1^)	0.047	0.047	0.049	0.058
OM digestibility (g/kg) ^4^		504	515	535	564

^1^ Net energy of lactation, determined from accumulated gas production at 24 h (mL/200 mg DM) and ether extract content, as described by [38]. ^2^ In vitro following the Menke and Steingass [36] procedure. ^3^ According to the model of Ørskov and McDonald [37]. ^4^ Apparent, in whole tract, according to Menke and Steingass [36].

### 3.1. Autumn Calving Season

During confinement, the OD-GRZ cows had a 46% lower likelihood of eating during the first 90 min than the CB-GRZ cows did (*p* < 0.01), they tended to have a 24% lower probability of lying at confinement (*p* = 0.06), they were 65% more likely to be standing during the total time (*p* = 0.05) and they had triple the probability of standing in the first 90 min at confinement (*p* < 0.01, Table 3). 

During grazing, both treatments had similar first grazing meal lengths (*p* = 0.29, Table 4), but the OD-GRZ cows tended (*p* = 0.09) to have a higher bite rate in this period (GM_1_), and they had a higher bite rate (+16%, *p* < 0.01) during the rest of the time in the paddock (GM_0_). The probability of the cows eating during the first 90 min at pasture was 42% higher in the OD-GRZ cows (*p* < 0.01), while their probability of ruminating during the first 90 min was 4 times lower compared to the CB-GRZ cows (*p* < 0.01). There was no effect of the level of environmental exposure on the lying time during grazing, but the CB-GRZ cows were 7 times more likely to stand during the first 90 min at pasture (*p* < 0.01, Table 4).

For ACS, neither the total nor pasture DM intake differed among the treatments, although the CB-TMR cows were ~20% more efficient in converting feed into milk than the MS cows, expressed as kg FPCM/kg DM (*p* = 0.03) or MCal NEl milk/Mcal of diet NEl (*p* = 0.02, Table 5). 

The ruminal microbial CP output was 32% higher in the CB-TMR cows than in the MS cows *(p* < 0.01), with no differences in grams of CP per kg digestible OM (Table 6). 

The CB-TMR cows had a 35% higher milk yield (*p* = 0.02), 32% higher energy retained in the milk (*p* = 0.03) and 33% and 35% higher protein and lactose yields (kg/cow/day) compared to the MS cows *(p* < 0.01) without differences in milk composition (Table 7).

### 3.2. Spring Calving Season

During confinement, the probability of standing was the only variable that differed between the treatments (*p* = 0.03), with it being 29% higher in the OD-GRZ cows compared to the CB-GRZ cows (Table 3). However, during grazing, the OD-GRZ cows spent 24 min less time eating at the first grazing meal (*p* < 0.01) at a bite rate that was 13% higher during GM_1_ and 29% higher during GM_0_ *(p* < 0.01). In addition, during the first 90 min at pasture, the OD-GRZ cows had a higher probability of ruminating *(p* < 0.01), accompanied by a lower probability of standing *(p* < 0.01) and a higher probability of lying (*p* = 0.05, Table 4). 

There was no effect of the feeding system nor environmental exposure on the pasture and total DM intake, feed efficiency (Table 5) and microbial CP output (Table 6). 

For the SCS cows, the CB-TMR cows had a 20% higher milk yield *(p* < 0.01) and 27% energy retained in the milk *(p* < 0.01), and they had higher fat, protein and lactose yields (kg/cow/day) compared to the CB-GRZ and OD-GRZ cows *(p* < 0.01, Table 7), although no differences in the milk component contents were observed. 

## 4. Discussion

### 4.1. Weather Conditions during Both Calving Seasons

The daily mean temperature during the sampling weeks were within the historical values for these periods of the year. However, the monthly accumulated rainfall levels were about 50% below the historical averages (Instituto Uruguayo de Meteorología [49]). Thus, it is clear that the cows experienced relatively benign weather during the sampling weeks, with no heavy or accumulated rainfall or extreme heat (i.e., daily THI mean above 72) in either experiment. 

### 4.2. Autumn Calving Season

The drier weather experienced by the autumn-calved cows prevented mud formation in the pens (feeders and/or rest area) of the OD-GRZ cows and/or heat stress, which would have been detrimental to the cows’ well-being and performance [5,7,8,50,51]. This, combined with good infrastructure design and maintenance in the feeding and resting areas of the OD-GRZ cows, likely explains why the CB-GRZ and OD-GRZ cows did not differ in most of the measured variables. However, the behavioral differences suggest that the conditions for the expression of behaviors (i.e., a lesser lying and a greater standing likelihood) were not optimal for the OD-GRZ cows, meaning that they likely adapted to compensate during the grazing period, as evidenced by the CB-GRZ cows spending more time on other activities (i.e., ruminating and standing) than the OD-GRZ cows during the first 90 min of grazing. Contrary to expectations, the OD-GRZ cows were not more likely to lie down and/or ruminate during grazing as a way to compensate for the lower rest time in confinement [5,8,12,28]. It seems clear that with the benign weather conditions, the time that the cows were able to lie down and ruminate during confinement was enough to fulfill their needs, while the energetic requirements of standing caused greater hunger/motivation to eat when arriving at pasture [52], thereby achieving similar pasture DMI and MCP outflows as the CB-GRZ cows. This also suggests that the grazing behavior differences were not predictors of productive outcomes for the CB-GRZ and OD-GRZ cows.

The CB-TMR cows had a higher (~35%) milk yield than the MS cows, whose diet (DM) consisted of 58% MR and 42% directly harvested pasture. The combination of TMR and pasture that had to be harvested by the cows implies a deferred contribution of diet components to the intake in these limited periods of time and a higher energy demand associated with walking and grazing activity, which could have impacted the productive outcome compared to the cows fed TMR alone. Fajardo et al. [53] observed that a 28% inclusion of grazed grass in the diet decreased the total DMI (20.0 kg DM/day) compared to open-door TMR (26.1 kg DM/day), which resulted in a 10% lower milk production (33.7 vs. 37.2 L/cow) and a more negative energy balance for the MS [54]. Jasinsky et al. [55] studied cow performance in open-door TMR and MS with 30% (DM) direct-harvested pasture included in the diet, and they noted a trend towards differences in milk production (28.1 vs. 26.3 L/cow/day), and there were no differences in the solids content, but it was accompanied by a higher level of energy retained in the milk and tissues in the TMR-treated cows. Salado et al. [22] observed that cows consuming diets with 21, 44 and 70% inclusions of direct-harvested pasture produced 6.5%, 20.4% and 27.6% less milk than cows in TMR systems (32.1, 28.4 and 26.8 vs. 34.2 L/cow/day, with a DMI of 22.4, 21.0 and 19.7 vs. 24.1 kg DM/cow/day, respectively). Their linear regression analysis showed an increase of 0.7 kg DM/cow/day and 1.1 L/cow/day for each 10% increase in TMR proportion in the diet [22]. In our study, as previously mentioned, the MS group had a 42% pasture inclusion in the diet, but a higher gap in milk production occurred with respect to the confined system, and there were higher absolute milk production values in all the treatments in all the studies, although with similar DMI levels. The higher milk yield of the confined cows was associated with a ~32% higher MCP outflow from the rumen and a ~18% increase in feed conversion efficiency (milk NE/diet NE) compared to the MS group. While the nutritional value of the pastures was lower than that of the mixed diet (as evidenced by the gas production at 24 and 30 h of incubation, the potentially degraded OM fraction, the rate of OM disappearance, the OM digestibility and the energy content), the pasture inclusion could have resulted in a lower intake of DOM and therefore a lower MCP. This, together with the unsynchronized supply of protein and energy and, on the other hand, the likely higher energy requirements for walking and grazing in the MS group, resulted in a lower milk performance and feed efficiency compared to the confined-system cows. 

### 4.3. Spring Calving Season

The average temperature was thermoneutral [56]. Although 19% of the early afternoon time was above a THI of 72, other times with lower values may have allowed for the OD-GRZ cows to dissipate their body heat load (i.e., below 20 °C, nighttime recovery) without impairing the cow’s performance compared to the CB-GRZ cows [19,57], which were allocated in better housing conditions during confinement time. Notwithstanding the higher FGML and total number of bites (6672 vs. 6210; not statistically analyzed) in the CB-GRZ group during the first grazing meal, the OD-GRZ cows had a higher probability of lying and ruminating and spending less time standing during the first 90 min at pasture. This is consistent with Pons et al. [28], who evaluated similar treatments but during summer when extreme environmental situations occurred, and they observed that OD-GRZ cows were less likely to be lying during confinement and were more likely to be lying in the paddock than CB-GRZ cows, which spent more time grazing. Although the OD-GR cows had a lower probability of standing during confinement, this was not accompanied by a higher probability of lying down, suggesting that the cows were reluctant to perform either of these behaviors, probably because the soil surface was not comfortable to lie on [51,58]. Lying deprivation during confinement resulted in the cows attempting to recoup lost resting time at pasture [5], thus leading to more intense grazing periods in an attempt to maximize resting time.

The confined cows had a similar intake to the MS cows, as well as a similar MCP outflow and MCP synthesis efficiency, although the TMR-CB cows produced 20% more milk than the MS cows. Swanepoel et al. [48] also found no correlation between the MCP flow from the rumen and milk production during early lactation. The cows in SCS (all treatments) started lactation with a BCS below that desired (2.78 ± 0.218) according to Roche et al. [59], which seems a frequent issue in MS SCS as a likely consequence of low pasture availability and quality during late lactation in late summer/early autumn [60]. As a result, the milk synthesis relied more on nutrients from the diet (~4.1% BW DMI) and less on nutrients from body reserves (¼ point of BCS mobilized in 50 days [13]), which diluted the efficiency of converting the feed into milk relative to the ACS cows, who had a higher feed efficiency at a DMI of ~3.4% BW, sustained by a drop of ½ a point of BCS in 40 days (BCS at calving: 3.05 ± 0.265).

The CB-TMR cows had a higher milk yield vs. both mixed systems, with no impact on milk components. White et al. [24] reported low milk responses in TMR cows vs. MS cows during spring, although the absolute values were lower than in our study. Although the differences between the feeding systems in this period responded to a 56% pasture inclusion in the diet of the MS cows, the productive gap with TMR feeding resembled that reported by Salado et al. [22] between TMR and MS with a pasture inclusion in the diet of 44% and that reported by Bargo et al. [61] with a 30% pasture inclusion in the diet. According to Salado et al.’s [22] regression analysis, with the actual pasture inclusion levels, our MS cows should have ingested almost 4 kg total DM less than those in the TMR system, which did not occur. The MCP outflow was not affected by the feeding system, and the TMR fed cows were only 8% more efficient at converting feed into milk (milk NE/diet NE) than the MS cows despite the high pasture inclusion in the MS diet. This could be due to the similar pasture and TMR nutritional value as well as the total DMI, with a possibly similar total digestible nutrient input for microbial growth in all treatments, as found by Mendoza et al. [62] and Pastorini et al. [63]. As part of the consumed energy was directed to incremental maintenance functions such as walking and grazing activity in the MS cows compared to the TMR-fed cows [55,64], a lower milk yield and feed efficiency was achieved.

## 5. Conclusions

No significant differences were observed between MS cows subjected to different levels of environmental exposure in terms of milk yield, feed efficiency, DMI or MCP in any calving season. The results demonstrate that under non-detrimental environmental conditions (i.e., no frequent heavy rains or severe heat waves), well-managed outdoor soil-bedded milk production systems can achieve the same high milk production potential as improved infrastructure systems (i.e., compost-bedded pack barns with cooling capacity). Notwithstanding, the observed differences in behavior between the treatments during confinement and grazing suggest that the OD-GRZ cows may have resorted to adaptive mechanisms to compensate for the worse conditions to express their behavior during confinement.

The autumn-calved TMR-fed cows had a substantially higher (~35%) milk yield with no impact on milk component levels vs. both mixed systems, regardless of environmental exposure. This was associated with a higher MCP outflow from the rumen (~32%) and an ~18% increase in milk NE/diet NE, which was likely due to the higher nutritional value of the TMR diet compared to the pasture. The spring-calved TMR-fed cows also had a higher milk yield vs. both mixed systems, with no impact on milk component levels regardless of environmental exposure. However, the MCP outflow was not impacted, and the TMR-fed cows were only 8% more efficient at converting feed into milk than the MS cows (milk NE/diet NE), which was likely due to the similar total DMI as well as the pasture’s and TMR’s nutritional value. 

Further research should focus on measuring ‘hidden costs’ not evaluated in this study, such as impaired energy–metabolic status and immune functions, which could impact reproductive efficiency and long-term variables like the lifetime productivity of mixed systems subjected to different environmental exposures.

## Figures and Tables

**Table 3:** Confinement behavior of autumn- and spring-calved cows with low (CB-GRZ) or high (OD-GRZ) environmental exposure.

				CB-GRZ	OD-GRZ	SEM	*p*-Value
**Autumn calving**							
	Probability of						
		Eating	90 min	0.82	0.56	0.039	<0.01
			Total	0.43	0.37	0.036	0.25
		Ruminating	90 min	0.01	0.03	0.012	0.19
			Total	0.16	0.21	0.027	0.20
		Lying	90 min	0.01	0.01	0.012	0.90
			Total	0.33	0.25	0.023	0.06
		Standing	90 min	0.10	0.33	0.022	<0.01
			Total	0.20	0.33	0.038	0.05
**Spring calving**							
	Probability of						
		Eating	90 min	0.79	0.77	0.061	0.84
			Total	0.25	0.25	0.027	0.99
		Ruminating ^1^	90 min	-	-	-	-
			Total	0.36	0.38	0.016	0.36
		Lying	90 min	0.02	0.02	0.010	0.52
			Total	0.48	0.51	0.023	0.27
		Standing	90 min	0.14	0.15	0.059	0.90
			Total	0.27	0.21	0.013	0.03

^1^ Did not converged due to insufficient number of observations.

**Table 4:** Grazing behavior of autmn- and spring-calved cows with low (CB-GRZ) or high (OD-GRZ) environmental exposure.

			CB-GRZ	OD-GRZ	SEM	*p*-Value
**Autumn calving**						
FGML (min)^1^			84	71	9.1	0.29
BR (bites/min)^2^		GM_1_	45	50	1.7	0.09
		GM_0_	44	51	1.1	<0.01
Probability of						
	Eating	90 min	0.55	0.78	0.044	<0.01
		Total	0.45	0.52	0.034	0.18
	Ruminating	90 min	0.33	0.08	0.025	<0.01
		Total	0.31	0.25	0.017	0.03
	Lying	90 min	0.23	0.12	0.049	0.19
		Total	0.41	0.39	0.040	0.74
	Standing	90 min	0.14	0.02	0.025	<0.01
		Total	0.10	0.06	0.016	0.12
**Spring calving**						
FGML (min) ^1^			139	115	6.1	<0.01
BR (bites/min) ^2^		GM_1_	48	54	1.5	<0.01
		GM_0_	41	53	1.8	<0.01
Probability of						
	Eating	90 min	0.90	0.89	0.017	0.69
		Total	0.56	0.63	0.022	0.06
	Ruminating	90 min	0.01	0.05	0.012	0.01
		Total	0.17	0.23	0.013	0.01
	Lying	90 min	0.01	0.08	0.032	0.05
		Total	0.19	0.26	0.035	0.22
	Standing	90 min	0.04	0.00	0.011	<0.01
		Total	0.18	0.07	0.014	<0.01

^1^ FGML = first grazing meal length. ^2^ BR = bite rate. GM_1_ = grazing meal 1, considered as first 85 min for ACS and 140 min for SCS. GM_0_ = all scan samplings not considered in GM_1_.

**Table 5:** Intake and efficiency of autumn- and spring-calved cows in confined (CB-TMR) and mixed systems with low (CB-GRZ) or high (OD-GRZ) environmental exposure.

				CB-TMR	CB-GRZ	OD-GRZ	SEM	*p*-Value
**Autumn calving**								
	Intake							
		Pasture (kg DM/cow/day)		-	9.3	9.5	1.29	0.94
		Total						
			(kg DM/cow/day)	24.6	22.4	22.6	1.08	0.33
			% BW	3.44	3.31	3.27	0.12	0.60
	Efficiency							
		kg FPCM/kg DM ^1^		1.70 ^a^	1.41 ^b^	1.40 ^b^	0.07	0.02
		NE Milk/NEl diet		0.77 ^a^	0.66 ^b^	0.65 ^b^	0.03	0.03
**Spring calving**								
	Intake							
		Pasture (kg DM/cow/day)		-	15.4	13.9	2.01	0.61
		Total						
			(kg DM/cow/day)	28.8	27.0	25.5	1.73	0.43
			% BW	4.39	4.08	3.93	0.24	0.42
	Efficiency							
		kg FPCM/kg DM ^1^		1.33	1.19	1.23	0.09	0.49
		NE Milk/NEl diet		0.62	0.57	0.58	0.04	0.58

^a,b^ Means within season with different superscripts differ (*p* < 0.05). ^1^ FPCM = fat–protein-corrected milk, according to FAO [32].

**Table 6:** Microbial crude protein synthesis of autumn- and spring-calved cows in confined (CB-TMR) and mixed systems with low (CB-GRZ) or high (OD-GRZ) environmental exposure.

			CB-TMR	CB-GRZ	OD-GRZ	SEM	*p*-Value
**Autumn calving**							
	Creatinine	(mg/L urine)	989 ^b^	1211 ^a^	908 ^b^	47.9	<0.01
	Allantoine	(mg/L urine)	2960 ^a^	2924 ^a^	2442 ^b^	136	0.02
	Microbial Crude Protein						
		(g CP/cow/day)	1840 ^a^	1304 ^b^	1480 ^b^	80	<0.01
		(g CP/kg DOM/day) ^1^	134	115	127	6.4	0.12
**Spring calving**							
	Creatinine	(mg/L urine)	687 ^a^	519 ^b^	609 ^ab^	31.1	<0.01
	Allantoine	(mg/L urine)	2762 ^a^	2106 ^b^	2286 ^b^	115	0.01
	Microbial Crude Protein						
		(g CP/cow/day)	2169	2167	1905	110	0.21
		(g CP/kg DOM/day) ^1^	144	161	146	9.0	0.20

^a,b^ Means within season with different superscripts differ (*p* < 0.05). ^1^ DOM = digestible organic matter.

**Table 7:** Milk yield per cow of autumn- and spring-calved cows in confined (CB-TMR) and mixed systems with low (CB-GRZ) or high (OD-GRZ) environmental exposure.

			CB-TMR	CB-GRZ	OD-GRZ	SEM	*p*-Value
**Autumn calving**							
	L/cow/day		42.8 ^a^	31.9 ^b^	31.7 ^b^	2.39	0.02
	kg FPCM ^1^/cow/day		42.1 ^a^	32.2 ^b^	32.0 ^b^	2.53	0.03
	Mcal NE/cow/day		31.7 ^a^	24.1 ^b^	24.0 ^b^	1.86	0.03
	Fat	g/kg	38.1	38.3	40.0	0.148	0.72
		kg/day	1.64	1.26	1.28	0.116	0.08
	Protein	g/kg	33.5	33.2	33.0	0.067	0.89
		kg/d	1.43 ^a^	1.09 ^b^	1.06 ^b^	0.069	<0.01
	Lactose	g/kg	51.0	50.1	50.3	0.063	0.59
		kg/d	2.19 ^a^	1.64 ^b^	1.61 ^b^	0.113	<0.01
**Spring calving**							
	L/cow/day		40.7 ^a^	34.1 ^b^	33.7 ^b^	1.15	<0.01
	kg FPCM ^1^/cow/day		38.5 ^a^	30.6 ^b^	30.2 ^b^	1.35	<0.01
	Mcal NE/cow/day		28.4 ^a^	22.4 ^b^	22.3 ^b^	1.03	<0.01
	Fat	g/kg	34.4	34.2	32.00	0.120	0.34
		kg/d	1.44 ^a^	1.14 ^b^	1.09 ^b^	0.060	<0.01
	Protein	g/kg	30.3	29.6	30.1	0.052	0.70
		kg/d	1.27 ^a^	1.00 ^b^	1.02 ^b^	0.051	<0.01
	Lactose	g/kg	49.0	47.8 ^y^	49.4 ^x^	0.044	0.06
		kg/d	2.05 ^a^	1.61 ^b^	1.68 ^b^	0.079	<0.01

^1^ Fat–protein-corrected milk, according to [32]. ^a,b^ Means within season with different superscripts differ (*p* < 0.05). ^x,y^ Means within season with different superscripts tend to differ (*p* < 0.10).

## Data Availability

Data are available upon request to the corresponding author.

## Supplementary Materials

The following supporting information can be downloaded at: https://www.mdpi.com/article/10.3390/ani14131905/s1, Table S1: Meteorological conditions in autumn (ACS) and spring (SCS) calved season cows during sampling periods.
