# Supplementary material for: Behavior, Intake, Digestion and Milk Yield of Early Lactation Holstein Dairy Cows with Two Levels of Environmental Exposure and Feeding Strategy
Source: Animals (Basel). 2024 Jun 27;14(13):1905. doi: 10.3390/ani14131905 (PMC11240589; doi:10.3390/ani14131905)
Supplement: Supplementary file 1 [file animals-14-01905-s001.zip › animals-3056109-supplementary.pdf]

animals

MDPI

Article

Behavior, Intake, Digestion and Milk Yield of early lactation Holstein dairy cows with two levels of environmental exposure and feeding strategy

Maria Noel Méndez [1]*, Nadia Swanepoel [2], Peter H. Robinson[2], Victoria Pons[1], Alejandra Jasinsky[1], Maria de Lourdes Adrien[1] and Pablo Chilibroste [3]

[1] Departamento de Ciencias Veterinarias y Agrarias, Facultad de Veterinaria, Universidad de la República, Paysandú 60000, Uruguay

[2] Department of Animal Science, University of California, Davis, CA 95616, USA

[3] Departamento de Producción Animal y Pasturas, Facultad de Agronomía, Universidad de la República, Paysandú 60000, Uruguay; pchili@fagro.edu.uy

* Correspondence: noemp21@gmail.com

Supplementary Table S1.

Supplementary Table S1. Meteorological conditions in autumn (ACS) and spring (SCS) calved cows during sampling periods[1]

	ACS		SCS	
	**Value**	**SEM**	**Value**	**SEM**
Daily Mean Temperature (ºC)	16	1.4	19	2.9
Relative Humidity (%)	86	9.5	61	5.1
Temperature Humidity Index[2]				
Daily Mean	61	2.3	64	4.1
% day < 68	99	3.2	69	14.9
% day 68 - 72	1	3.2	13	4.4
% day > 72	0	0.0	19	17.5
Rain (mm) sampling period	12	-	2	-
monthly accumulation	71	-	37	-
Wind speed (km/h)	3	2.7	4	3.4

[1]Average data of 5 milk recording days + 2 days previous. [2]According to Armstrong [31].
